# Size-Controlled Preparation of Docetaxel- and Curcumin-Loaded Nanoemulsions for Potential Pulmonary Delivery

**DOI:** 10.3390/pharmaceutics15020652

**Published:** 2023-02-15

**Authors:** Azren Aida Asmawi, Norazlinaliza Salim, Emilia Abdulmalek, Mohd Basyaruddin Abdul Rahman

**Affiliations:** 1 Integrated Chemical BioPhysics Research, Department of Chemistry, Faculty of Science, Universiti Putra Malaysia, Serdang 43400, Malaysia; 2 UPM-MAKNA Cancer Research Laboratory, Institute of Bioscience, Universiti Putra Malaysia, Serdang 43400, Malaysia; 3 Centre of Foundation Studies for Agricultural Science, Universiti Putra Malaysia, Serdang 43400, Malaysia

**Keywords:** docetaxel, curcumin, lipid-based carrier, nanoemulsion, pulmonary delivery, response surface methodology

## Abstract

Lung cancer is one of the deadliest pulmonary diseases in the world. Although docetaxel (DTX) has exhibited superior efficacy in lung cancer treatment, it has demonstrated numerous adverse effects and poor bioavailability. The natural product extract, curcumin (CCM), has reportedly reduced toxicity and synergistically improved DTX bioavailability. Nonetheless, the hydrophobic nature of DTX and CCM limits their clinical use. Nanoemulsion pulmonary delivery of DTX and CCM has demonstrated potential as a drug carrier to alleviate these drawbacks. The controlled preparation of inhalable DTX- and CCM-loaded nanoemulsions within the 100 to 200 nm range was explored in this study. A response surface methodology (RSM) based on a central composite design (CCD) was utilized to fabricate the desired size of the nanoemulsion under optimized conditions. Different process parameters were employed to control the size of the nanoemulsions procured through a high-energy emulsification technique. The size of the resultant nanoemulsions decreased with increasing energy input. The actual response according to the targeted sizes for DTX- and CCM-loaded nanoemulsion models exhibited excellent agreement with the predicted value at below 5% residual standard error under optimized conditions. The nanoemulsion of 100 nm particle size demonstrated better membrane permeability than their larger counterparts. Moreover, the formulations documented favorable physicochemical and aerodynamic pulmonary delivery properties and reduced toxicity in human lung fibroblast (MRC-5) cells. Hence, this tunable size of nanoemulsions could be a suitable alternative drug delivery for pulmonary diseases with increased local lung concentration.

## 1. Introduction

In 2020, lung cancer resulted in approximately 18% of cancer deaths, and this illness is one of the most pervasive cancers worldwide [1]. Limited access to prompt diagnosis and therapy due to the lack of early observable symptoms has resulted in the five-year survival rate for lung cancer becoming the lowest among all cancers [2,3]. Typically, the disease is only discernible at phases where the cancerous cells or tissues are numerous and widespread in the body, during which chemotherapy is a routine remedial recourse to curb the malignant tumor from spreading. Docetaxel (DTX) is commonly employed as a chemotherapeutic agent to treat small-cell lung cancer (NSCLC) [4,5,6]. However, its use is limited by dose-dependent toxicities in organ systems and low patient-response rates (<30%) due to less sensitivity and drug resistance [7,8]. Nonetheless, curcumin (CCM) has been reported to synergistically improve DTX anticancer activities by inhibiting tumor proliferation, enhancing tumor cell apoptosis, and reducing toxicity in normal cells [9,10].

The CCM is a substance extracted from the *Curcuma longa* turmeric rhizome and has gained interest in the anticancer field due to its medicinal attributes and potential as a chemosensitizer [11,12,13]. Chemosensitizers enhance the chemotherapeutic drugs’ therapeutic index by making the tumor cells more sensitive to the drugs. Chemosensitizers are multitargeting compounds with anti-inflammatory, antioxidant, and chemotherapeutic effects with insignificant toxicity when evaluated in animal models, even at high doses [14,15,16]. The findings suggest that CCM could be considered an attractive therapeutic drug if combined with DTX. Nevertheless, treatment effectiveness relies heavily on drug delivery strategies to the targeted site. DTX and CCM were found to be insufficiently soluble in aqueous media, hence, incorporating them into pharmaceutical products is an issue. Consequently, developing a viable drug delivery strategy that enhances the solubility and bioavailability of the compounds is necessary.

Pulmonary delivery of nanomedicine is currently among the most propitious drug delivery alternatives to the lungs via inhalation. Drug bioavailability in the lungs might be improved compared to oral and parenteral routes, as the administration excludes gastrointestinal metabolism and first-pass effects in the liver [17,18,19,20]. This alternative approach also provides direct drug depositions on the desired site [21,22,23]. Moreover, concentrated drug targeting possibly prolongs drug occupancy in pulmonary receptors, thus increasing the effective drug dosage and minimizing adverse side effects [21,24,25]. Nowadays, nanocarriers have attracted attention as delivery vectors to protect the drug from early discharge, aid cellular uptake, and boost maximum drug absorption with negligible toxicity [26,27,28]. Moreover, the lipid excipient accessibilities within safety standards and capabilities of nanocarriers to enhance hydrophobic drug solubility, long-term stability, ease of production, and deep-lung deposition capacity have revolutionized lipid-based systems, specifically nanoemulsions, as promising drug carrier systems for pulmonary delivery applications.

To date, only a few studies have been documented on drug encapsulations in nanoemulsion systems for pulmonary applications [21,24,29,30,31]. The inhalable rifampicin-loaded system was reported safe and possessed better cell internalization potential with a high drug content [21]. The observations agreed with reports claiming aerosolized tea tree oil nanoemulsions were capable of significantly relieving lung inflammation symptoms and injuries in fungal and bacterial pneumonia-infected rats without toxicity [32]. Several studies have also recorded improved inhalable drug-loaded nanoemulsion efficacy towards A549 lung cancer cell lines with sustained drug release in contrast to free drugs, thus reducing the amount of drug available for adverse effects [24,31]. Although pulmonary applications of nanoemulsion research are still in their infancy, inhalable CCM- and DTX-loaded nanoemulsion fabrications for synergistic effects in lung cancer treatments are feasible.

Several physicochemical and aerodynamic nanoemulsion system requirements necessitate consideration to achieve optimum deep-lung-region depositions where tumors are commonly detected. Tang et al. [33] and Wang et al. [34] discovered that moderate-sized nanoparticles (<200 nm) favored aggregation in tumor areas due to the improved permeability and retention (EPR) effect. Consequently, size is a crucial characteristic of most new drug carrier systems for tumor targeting via pulmonary delivery. Nanoemulsions are also thermodynamically unstable, and emulsification methods involving high and low energies could affect the stability, distribution, and nanoemulsion system droplet size [35,36,37,38]. Accordingly, the present study constructed an optimization strategy for DTX- and CCM-loaded nanoemulsion procurement utilizing a central composite design (CCD) in response surface methodology (RSM) to maximize or minimize the response based on the desired targets. This study provided deep insight and understanding of how the size of DTX- and CCM-loaded nanoemulsions interplay between different process parameters that affect their physicochemical property, aerosolization efficiency, stability, permeability, and toxicity.

## 2. Materials and Methods

### 2.1. Materials

Docetaxel (DTX, >98% purity) and Curcumin (CCM, >75% purity) were provided by Acros Organics (Fair Lawn, NJ, USA) and Merck (Darmstadt, Germany), respectively. Span 85, 3-[4,5-dimethylthiazol-2-yl]-2,5-diphenyltetrazolium bromide (MTT), α-tocopherol, fluorescein, dimethyl sulfoxide, and safflower seed oil were obtained from Sigma-Aldrich (Steinheim, Germany). Surfactants including lecithin Lipoid S75 and Tween 85 were obtained from Lipoid GmBH (Ludwigshafen, Germany) and Fluka Chemie GmbH (Buchs, Switzerland), respectively. Glycerol was obtained from J.T. Baker (Phillipsburg, NJ, USA), and palm kernel oil ester (PKOE) was synthesized via the enzymatic transesterification method [39]. The materials were all analytical grade and used without additional purification.

### 2.2. Preparation of Nanoemulsions

The DTX- and CCM-loaded nanoemulsion compositions were obtained from our previously optimized formulation [30]. Briefly, the nanoemulsions were fabricated using high-energy emulsification techniques. The oil phase was prepared by dissolving lecithin (2.50%, *w*/*w*) with a mixture of PKOEs and safflower seed oil at a 1:1 ratio (6.00%, *w*/*w*) at 45 °C for 30 min before mixing with the active ingredient and a mixture of Tween^®^ 85 and Span^®^ 85 at a 9:1 ratio (2.00%, *w*/*w*) for another 30 min. The mixture was then supplemented with 0.05% (*w*/*w*) of α-tocopherol and stirred for 5 min at 40 °C. The prepared oil phase was added dropwise into the aqueous phase, which contained glycerol (2.50%, *w*/*w*) and deionized water (86.95%, *w*/*w*) to form the pre-mixed emulsion. The resulting emulsion was subjected to a high-shear homogenizer for further homogenization.

### 2.3. Experimental Design

A Central Composite Design (CCD) in Response Surface Methodology (RSM) was used for the experimental design to examine the effects of process parameters on particle size and PDI. Thirty experiments for four factors were generated using Design-Expert^®^ software (Stat-Ease Inc., Minneapolis, MN, USA). These experiments included six repetitions of center points, eight axial points, and sixteen factorial points. The coding of each independent variable was determined from the preliminary study and is summarized in Table 1.

### 2.4. Statistical Analysis and Model Verification

The ANOVA was performed to study the significance of each term in the models. The correlation of coefficient (*R*^2^), *f*-values, and *p*-values for each significant term were determined. The generated models were then represented by quadratic polynomial equations and subjected to validation tests to determine their adequacy in predicting the response values by comparing them with the experimental values. Recommended optimum process parameters for targeted nanoemulsion sizes of 100, 150, and 200 nm were conducted to validate the response values predicted by the model. The following equation was used to determine the percentage of residual standard error (RSE) for each response.

RSE (%) = [(Experimental value − Predicted value)/Predicted value] × 100(1)

### 2.5. Physicochemical Characterization

#### 2.5.1. Particle Size Distribution and ζ-Potential Determination

The optimized nanoemulsions containing 2.0 mM DTX and CCM with targeted sizes 100, 150, and 200 nm were prepared using the method described in Section 2.2. The mean particle size, polydispersity index (PDI), and ζ-potential of the samples were measured using Zetasizer (Nano ZS90, Malvern Instruments, Worcestershire, UK). The sample was diluted 1000-fold using deionized water and loaded into a zeta potential plastic cuvette until full. The cell area temperature was stabilized by equilibration for 2 min at room temperature (25 ± 1 °C) before measurements. The results were averaged over three experiments.

#### 2.5.2. Viscosity Measurement

A viscometer (Brookfield DV-II+, Middleboro, MA, USA) with spindle SC4-18 was used to measure the viscosity of the formulated nanoemulsions. The viscosity was measured in centipoise (cP) with a rotary transducer based on the deflection of the calibrated spring. The measurements were conducted at room temperature (28 ± 1 °C) and repeated in triplicate.

#### 2.5.3. pH Measurement

The pH of nanoemulsions was measured using a pH meter (Delta 320, Mettler Toledo, Tokyo, Japan). Prior to usage, the pH meter was calibrated using pH 4, 7, and 10. The glass probe was rinsed with deionized water before being directly submerged in the sample without dilution. The measurement was then carried out three times at room temperature (28 ± 1 °C) and the mean pH value of the sample was recorded.

#### 2.5.4. Entrapment Efficiency

The entrapment efficiency was determined by the ultrafiltration method [40] using a Centrisart tube with a molecular weight cut-off of 10 kDa (Sartorius, Gottingen, Germany). Centrifugation was performed on the device containing 2 mL of the sample in the donor chamber for 30 min at 6000 rpm to separate the unloaded DTX or CCM in the aqueous phase into the inner floater tube. The drug solution in both the recovery and donor chambers was collected and quantified using the HPLC method as outlined in our previous report [30]. The following equation was used to calculate the percentage of drug that was entrapped.

(2) $$\mathrm{Entrapment}\;\mathrm{efficiency}\;\left(\%\right)=\frac{\mathrm{Winitial}\;-\;\mathrm{Wobtained}\;}{\mathrm{Winitial}}\;\times \;100\;$$

where “Winitial” is the amount of drug loaded in the formulation, while “W_obtained_” is the amount of unloaded drug in the aqueous phase of the formulation.

### 2.6. Aerosolization and Inhalation Studies

#### 2.6.1. Nebulizer Aerosol Rate and Output Determination

An OMRON MicroAIR nebulizer (NE-U22V1, Kyoto, Japan) was filled with 3.0 mL of 2.0 mM DTX- or CCM-loaded nanoemulsion samples. The samples were nebulized to dryness at a vacuum-assisted constant flow rate of 15 L/min as outlined by the European Standard (EN ISO 27427) for nebulizers [41]. The flow rate represents human inspiratory flow rates for a mid-inhalation flow rate of an adult patient. The nebulizer was weighed before and after nebulization to determine the residual volume of the solution in the nebulizer. The time required for the sample to reach dryness was also recorded. The aerosol output and rate were calculated using the following equations.

Aerosol output (%) = [(W_initial_ − W_left_) /W_initial_] × 100 (3)

Aerosol rate (g/min) = [(W_initial_ − W_left_/*t*] × 100 (4)

where W_initial_ is the weight of the nebulizer containing the sample before nebulization, W_left_ is the weight of the nebulizer after nebulization, and *t* is the time needed to nebulize the sample to dryness.

#### 2.6.2. Laser Diffraction Analysis

The effect of the aerosol rate on the aerosolization properties of nebulized nanoemulsions was measured using a Spraytec laser diffraction system (Malvern Instruments Ltd., Worcestershire, UK) equipped with an extraction accessory. A nebulizer containing 3 mL of 2.0 mM DTX- or CCM-loaded nanoemulsion was placed perpendicularly to the laser lens axis at 2 cm away from the laser beam. The nanoemulsion was then nebulized at a flow rate of 15 mL/min. The intensity of light scattered as a laser beam that passes through the nanoemulsion aerosols was then measured and analyzed using RTSizer Software (version 5.51, Malvern Instruments Ltd., Worcestershire, UK). The value of Dv_10_ (10% undersize particle distribution curve), Dv_50_, also known as the volume median diameter (VMD, 50% of the undersized particle distribution curve), Dv_90_ (90% undersize particle distribution curve), volume-weighted mean diameter (D_[4,3]_), surface area-weighted mean diameter (D_[3,2]_), and span, which measures the distance between 10% and 90% points, were all calculated and normalized with the midpoint. The results were averaged over three experiments.

### 2.7. Stability Study

The stability study under different storage conditions was carried out based on the method described by Syed Azhar et al. [42]. Three test tubes containing 2.0 mM DTX- and CCM-loaded nanoemulsion samples were kept at 5 ± 2 °C and 60 ± 5% relative humidity (refrigerator), 29 ± 1 °C and 50 ± 5% relative humidity (room temperature), and 45 ± 2 °C and 50 ± 5% relative humidity in an incubator (DK-S1020, DAIKI Sciences Co., Ltd., Seoul, Republic of Korea) for over three months. The particle size and PDI values were determined for the three temperatures. The physical appearance of the formulations was observed and recorded as well. The formulation must show insignificant changes to classify them as stable during storage. The possible destabilization mechanism that could occur during storage was determined by plotting the graph of (1/*r*^2^) vs. time for coalescence and *r*^3^ vs. time for Ostwald ripening, where *r* is the mean of particle size.

### 2.8. Drug Permeation Studies

#### 2.8.1. Permeability Study in Zebrafish Embryo

The permeability test was performed on zebrafish (*Danio rerio*) embryos due to their suitability to perform imaging via microscopy [26]. The embryos were purchased from Danio Assay Laboratories Sdn. Bhd., Malaysia. Briefly, 12 viable fertilized embryos at 1 h of post-fertilization (hpf) with the optimum shape and absence of abnormalities were placed in a 96-well plate (one embryo/well) containing 100 μL of embryo media with 0.1% DMSO. The embryos were exposed to 100 μL of 100 ug/mL of fluorescein dye (positive control) and 100 μL of the nanoemulsion formulation containing 100 μg/mL of fluorescein with a particle size of 100, 150, and 200 nm, at 1, 6, and 24 h of post-exposure (hpe). Fluorescein was used as a fluorescence marker, which preferentially partitions into the lipophilic core of the nanoemulsion system. After exposure, the embryos were washed thoroughly with embryo media and visualized under an inverted fluorescence microscope, eclipse T*i*2 (Nikon, Tokyo, Japan) equipped with NIS Element software.

#### 2.8.2. Drug Release Study via Franz Diffusion Cell

In vitro DTX and CCM release from the nanoemulsion system utilizing the Franz diffusion cell method was performed using simulated lung fluid (SLF) at pH 7.4 containing 0.2% (*w*/*v*) Tween 80 as the dissolution media [43]. A cellulose acetate membrane with a pore size of 0.45 μm (Sartorius, Gottingen, Germany) covered with 300.0 μL of the 1.0% mucin solution simulating the mucus layer accumulated on the lung epithelium was used to separate the donor compartment from the acceptor compartment [44]. The drug release was carried out by loading 2.0 mL of 2.0 mM DTX- or CCM-loaded nanoemulsion into the donor compartment, while the acceptor compartment was filled with dissolution media. The experiment was performed under continuous stirring at 37 ± 1 °C. In order to maintain the sink condition, 0.5 mL of the sample aliquot was taken out and replaced with an equivalent volume of fresh dissolution media at predetermined intervals. The drug concentration in the collected samples was then analyzed using the HPLC method as described in our previous report [30].

### 2.9. In Vitro Nanotoxicity Assessment

The nanotoxicity profile of nanoemulsion formulations was assessed using 3-[4,5-dimethylthiazol-2-yl]-2,5-diphenyltetrazolium bromide (MTT) assay on the MRC-5 cell line using a modified method described by Kuen et al. [45]. Cells were seeded in 96-well plates at a 2.8 × 10^3^ cells/well density and allowed to grow for 24 h at 37 °C in a humidified incubator with 5% CO_2_. Prior to cellular treatment, the nanoemulsions containing 2.0 mM DTX and CCM were prepared and diluted using the RPMI complete medium containing 0.5% DMSO with drug concentrations ranging from 50.0 to 0.1 μM. The cell media were carefully discarded without disturbing the cells attached to the plate surface, and a volume of 100 µL of each sample concentration was pipetted into each well in triplicate and incubated for 48 h at 37 °C in a humidified incubator with 5% CO_2_. Approximately 0.5 mg/mL of the MTT solution (final concentration in each well) was added at the end of the incubation and was further incubated for 4 h. Following incubation, 80 µL of the solution in each well was carefully discarded without disturbing the formazan crystals, and 80 µL of DMSO was pipetted into each well to dissolve the crystals. An iMarkTM microplate reader (Bio-Rad, Hercules, CA, USA) was used to measure the MTT absorbance at 570 nm. The calculation for the cell viability percentage is shown below.

(5) $$\mathrm{Cell}\;\mathrm{viability}\;\left(\%\right)=\frac{A_{570}\;\mathrm{of}\;\mathrm{sample}\;}{A_{570}\;\mathrm{of}\;\mathrm{control}\;(\mathrm{cell}\;\mathrm{without}\;\mathrm{treatment})}\;\times \;100\;$$

### 2.10. Statistical Analysis

All data were analyzed using GraphPad Prism version 8.0.2 (Dotmatics, Boston, MA, USA) and presented as the mean and standard deviation. Statistical analysis was conducted using ANOVA to determine the significant difference between the test group. A statistically significant difference was defined as *p* < 0.05.

## 3. Results and Discussion

### 3.1. Model Fitting and Analysis of Variance

A Central Composite Design (CCD) in RSM was employed to optimize the independent variables in the process parameter, including the stirring rate (A) and speed (B) and homogenization rate (C), and speed (D), on the response of particle size and the polydispersity index (PDI). In the current study, the quadratic polynomial model best conformed to the experimental particle size results, while the linear polynomial model satisfied the experimental data of PDI values of the DTX- and CCM-loaded nanoemulsion formulations. Predicted particle size and PDI values in both formulations were obtained with regression Equations; Equations (6) and (7) for the DTX system and Equations (8) and (9) for the CCM-loaded nanoemulsion, as per the analysis of variance (ANOVA) experimental data-based suggestions. A positive coefficient equation indicates that the respective predictor variable is directly proportional to the response variable, hence, increasing parameter values would lead to rising response figures.

DTX-loaded nanoemulsion

Particle size = 175.10 − 9.50 A − 6.54 B − 31.23 C − 23.03 D − 3.06 AB + 3.26 AC + 1.73 AD − 4.63 BC + 7.89 BD + 0.81 CD − 9.23 A^2^ − 10.66 B^2^ − 0.80 C^2^ − 6.13 D^2^ (6)

PDI = 0.31 − 0.0028 A − 0.007 B − 0.046 C − 0.016 D(7)

CCM-loaded nanoemulsion

Particle size = 107.63 − 9.39 A − 8.20 B − 22.74 C − 20.42 D − 0.66 AB − 2.79 AC + 5.33 AD + 1.01 BC + 2.21 BD − 0.87 CD + 4.17 A^2^ + 4.05 B^2^ + 9.09 C^2^ + 8.19 D^2^ (8)

PDI = 0.27 − 0.011 A − 0.022 B − 0.068 C − 0.017 D(9)

**Figure 1:**
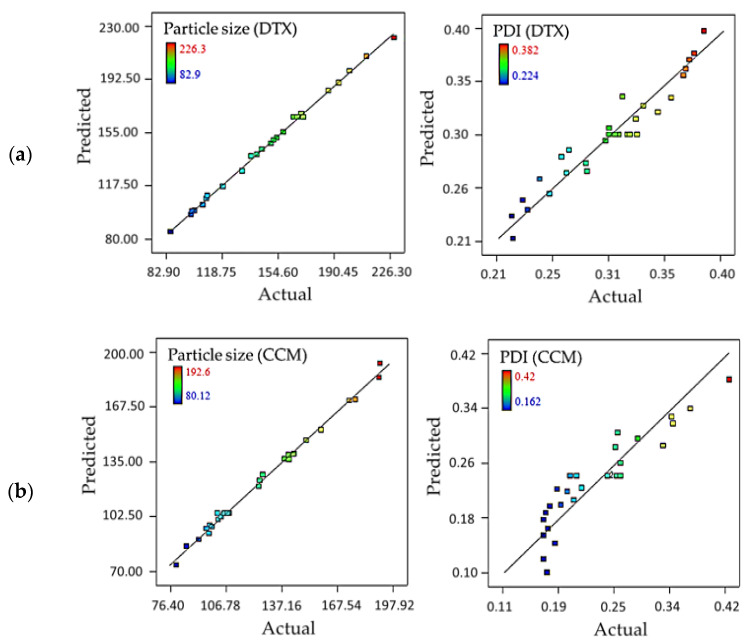
Scatter plot of predicted vs. actual particle size of (**a**) DTX-loaded nanoemulsion and (**b**) CCM-loaded nanoemulsion obtained from CCD experimental design. PDI: Polydispersity index.

The predicted and actual particle size linearity plot exhibited a well-fitting regression model compared to the PDI responses of the DTX and CCM systems (Figure 1). The particle size correlation coefficient (*R*^2^) values recorded by the DTX and CCM samples were 0.9983 and 0.9953, respectively, while 0.9288 and 0.8383 for PDI were documented. The values revealed that 99.83 and 99.53% of the particle size variability and 92.88 and 83.83% of the PDI variants were explainable by the regression models. The signal-to-noise (S/N) ratio for both the DTX and CCM formulations of particle size and PDI was more than 50 and 20, respectively. The S/N contrasts projected figures at design points to average prediction error. S/N numbers over 4 indicate a sufficient ratio and, therefore, could be employed to guide the design space.

The matched quadratic and linear models’ ANOVA results for particle size and PDI of the nanoemulsion systems constructed in the present study are summarized in Table 2. The mathematical models procured were significant and valid, where small *p*-values (<0.0001) and large *f*-values were recorded, stipulating a satisfactory fit between the regression models and experimental findings. Furthermore, pure errors, including experimental errors, were minimal as the lack of fit data were insignificant in both models. The stirring rate (A) and duration (B), and the homogenization rate (C) and duration (D) linear terms considerably affected the particle size of the DTX- and CCM-loaded nanoemulsions. Nevertheless, the interaction between CD terms was inconsequential. Other interactions, such as AB, AC, AD, BC, and BD, notably contributed to the DTX-loaded nanoemulsion particle size. Only a few interactions, namely, AC, AD, and BD, recorded significant relation to the particle size in the CCM formulation. The A^2^, B^2^, and D^2^ quadratic terms resulted in considerable implications on the particle size of the DTX-loaded nanoemulsion, while in the CCM formulation, all quadratic terms were significant. The regression coefficients of linear equations for PDI displaying B, C, and D documented a notable effect on the size distribution of DTX-loaded nanoemulsion, whereas all linear terms were significant in the CCM-loaded nanoemulsion.

### 3.2. The Central Composite Design Analysis

**Figure 2:**
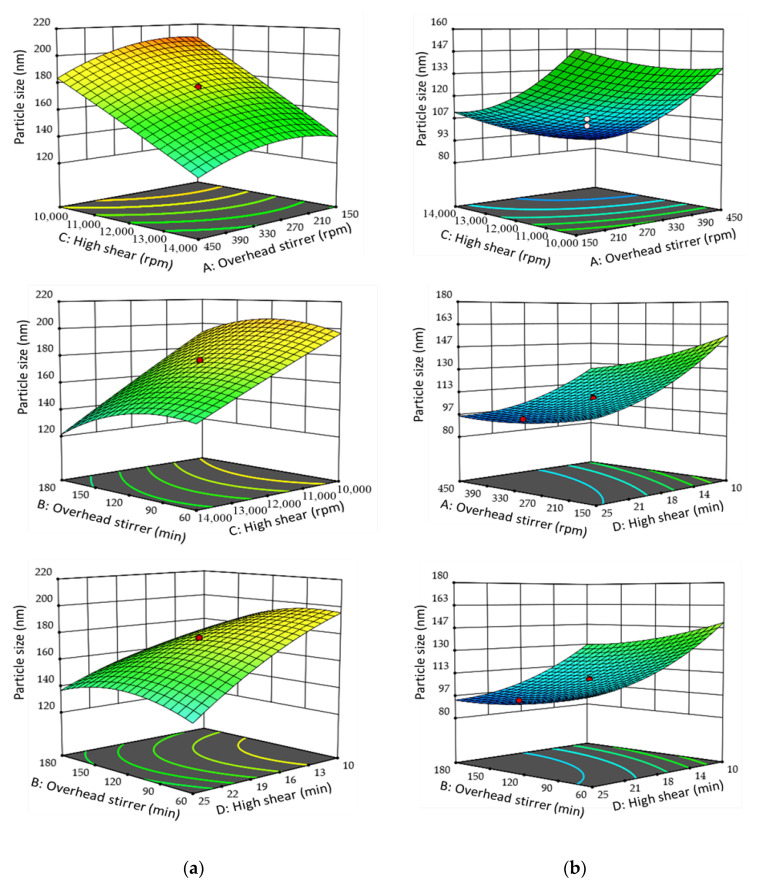
Three-dimensional response surface plot of (**a**) DTX-loaded nanoemulsion and (**b**) CCM-loaded nanoemulsion process parameters on the z-average hydrodynamic diameter (particle size). The models show the interaction between independent variables including A: Overhead stirrer speed (rpm); B: Overhead stirrer duration (min); C: High shear speed (rpm); D: High shear duration (min) toward the particle size.

The three-dimensional (3D) surface response model graphs and contour plots of the quadratic polynomial models are illustrated in Figure 2. The present study documented nanoemulsion particle size reduction upon stirring rate (from 150 to 450 rpm), stirring duration (from 60 to 180 min), and homogenization duration (from 10,000 to 14,000 rpm) increments in the DTX and CCM systems. The nanoemulsion particle size was substantially diminished from approximately 190 nm to 120 nm (DTX) and 130 to 84 nm (CCM) when the homogenization rate was over 11,000 rpm. The trend was attributed to the length of duration and amount of applied force to break and disperse the nanoemulsion particles into smaller sizes. However, the trend was reversed after the particles reached their minimum size at 13,000 rpm homogenization when utilizing high shear. The results implied that there was a threshold on process parameters for attaining the optimal effect. Consequently, no further particle size reduction was recorded after optimum dispersity was obtained at the particular power density. The overprocessing phenomenon could also explain the findings, wherein an intense homogenization process could lead to the recoalescence of the nanoemulsion particles into larger particles.

**Figure 3:**
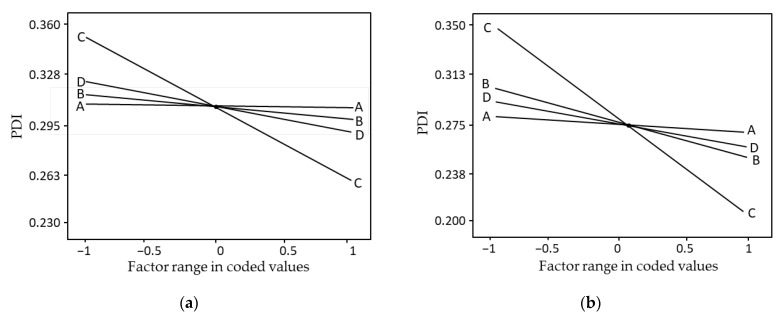
One factor plot of (**a**) DTX-loaded nanoemulsion and (**b**) CCM-loaded nanoemulsion on the response variable of PDI. A: Overhead stirrer speed (rpm); B: Overhead stirrer duration (min); C: High shear speed (rpm); D: High shear duration (min); PDI: Polydispersity index.

Under similar process parameters, the CCM-loaded nanoemulsion particles in this study were slightly smaller than the DTX counterpart. The differences might be attributable to the lower molecular weight of CCM (368.38 g/mol) compared to DTX (807.88 g/mol), which allowed minimum packing geometry. Furthermore, drugs with higher molecular weights could result in a viscous solution and reduced shear during emulsification, generating a larger nanoemulsion [46]. The results obtained in the present study were in good correlation with the findings reported by Fuentes et al. [47], as the mean particle size of nanoemulsion generally increases with an increase in the molecular weight of its composition [47]. Moreover, the one-factor plot of DTX- and CCM-loaded nanoemulsions exhibited a direct interaction between the process parameters and PDI value (Figure 3). The CCM-loaded nanoemulsion system documented a lower PDI value than DTX. The homogenization rate significantly contributed to decreased PDI, followed by homogenization duration, overhead stirrer duration, and overhead stirrer speed. A low PDI implies high kinetic stability, whilst high PDI results in a broader size range and low stability [42,48]. Hence, tailoring the nanoemulsion with a low PDI value is crucial to forming a stable system.

Although nanosized particles are advantageous for pulmonary delivery, different size ranges are critical to assess the efficacy of drug deposition in the targeted lung region. Furthermore, nanoemulsion size is closely related to its permeability. Particles under 200 nm have been demonstrated to possess good particle passage through cell membranes [49,50]. Therefore, several criteria for each factor were considered in this study to produce different targeted particle sizes (100, 150, and 200 nm) with low PDI values for effective pulmonary delivery. Small-sized and low PDI nanoemulsions produced at an optimal stirring rate and duration and the homogenization rate and duration could effectively diminish the overall cost and time to produce the nanoemulsion. The targeted size experimental results based on the RSM suggestions in this study are listed in Table 3. The actual value for particle size and size distribution when employing the suggested ideal conditions was in agreement with the predicted values. Moreover, the RSE percentage values were small, namely, <5%, which indicated that the generated models utilized in the prediction could prove their reliability in measuring the response of process parameters concerning particle size and PDI.

### 3.3. Physicochemical and Aerosolization Properties

The mean particle size of the formulated nanoemulsion in the present study was similar to the targeted particle size (100, 150, and 200 nm) as per the RSM projections (Table 4). Adding drug concentrations up to 2 mM to the systems did not significantly affect the particle size. However, the PDI values were increased with increasing particle size. The PDI values within the 0.18–0.26 range were documented by the 100 and 150 nm samples, suggesting a monodispersed suspension system. On the other hand, a PDI over 0.30 was recorded by the 200 nm nanoemulsion sample, indicating minimal polydispersion while still satisfactory for pulmonary application. Particle size, PDI, and ζ-potential are critical physicochemical properties when defining the stability of a nanoemulsion system. The stability of a nanoemulsion system would increase when its particle size is minimized. Moreover, Ostwald ripening could be reduced by setting a lower PDI to produce a highly homogenous distributed system [51]. In this study, all nanoemulsions exhibited zeta potential values over −30 mV, denoting an acceptable high negative surface charge to maintain stability. The stability can be increased by increasing the zeta potential value as it increased the degree of repulsion between the colloidal particles [38]. The relatively high charge of the formulated nanoemulsions of the current study was possibly affected by the anionic groups of fatty acids and glycol in oils and lecithin.

The nanoemulsion systems in this study exhibited neutral pH, 7.1–7.2, which was in the allowable range for pulmonary applications. The pH of a nanoemulsion formulation should closely resemble the pH of lung fluid. According to European Pharmacopeia, the pH of liquid mixtures for pulmonary administration purposes should be between 7.0 and 8.0 [52]. Furthermore, dispersion media pH alterations could initiate emulsion droplet coalescence resulting from electrostatic destabilization and disruption of the liquid film between the droplets. The drug entrapment efficiency documented by all nanoemulsion samples was found to be 100%, and both DTX and CCM were able to fully solubilize in their oil phase at a 2 mM concentration. Moreover, the viscosity of a nanoemulsion system increases with reduced particle size. The condition is attributable to water molecules entrapped in the system compared to the external phase with fewer water molecules. Smaller particle sizes would also promote particle-particle interactions and flow resistance from the high number of particles [53]. Consequently, viscosity critically influences the effectiveness of pulmonary drug delivery. High-viscosity formulations also necessitate more energy to be atomized into aerosols and contribute to increased aerodynamic diameter, hence reducing aerosolization efficiency [54,55].

The output and rate of aerosolization for all formulations were within the range of 98.3 and 99.1%, and 0.12 and 0.19 g/min, respectively, indicating low residual volumes left in the nebulizer and were nebulized at a high rate. The nebulization of larger particle nanoemulsions exhibited higher aerosol output and rate than the smaller particles. Large nanoemulsion particles possessing high electrical conductivity could produce high aerosol output when the electrostatic charge in the continuous phase or oil droplets was suppressed [24]. The condition prevented the oil droplets from sticking to the nebulizer surface and mesh holes, resulting in a high fraction of nanoemulsion aerosolization. The present study employed the laser diffraction method during the aerosolization stage to assess the summative inhalation aerosolization performance. Based on Table 4, the C200 formulation produced the smallest size of aerosol that exhibited particle aerodynamic diameters (DV_50_ = 4.63 ± 0.31 μm) and a relatively narrow size distribution (Span = 1.61 ± 0.18) followed by D200, C150, C100, D150, DC200, DC150, DC100, and D100. The results agreed with the influence of viscosity that was inversely proportional to the particle size. A decrease in viscosity led to declined flow resistance and subsequently increased nebulization efficiency. Consequently, small particle nebulized formulations were considered suitable in terms of the aerosol size range (<5 μm) for deep lung deposition by gravitational sedimentation.

### 3.4. Stability Evaluation

**Figure 4:**
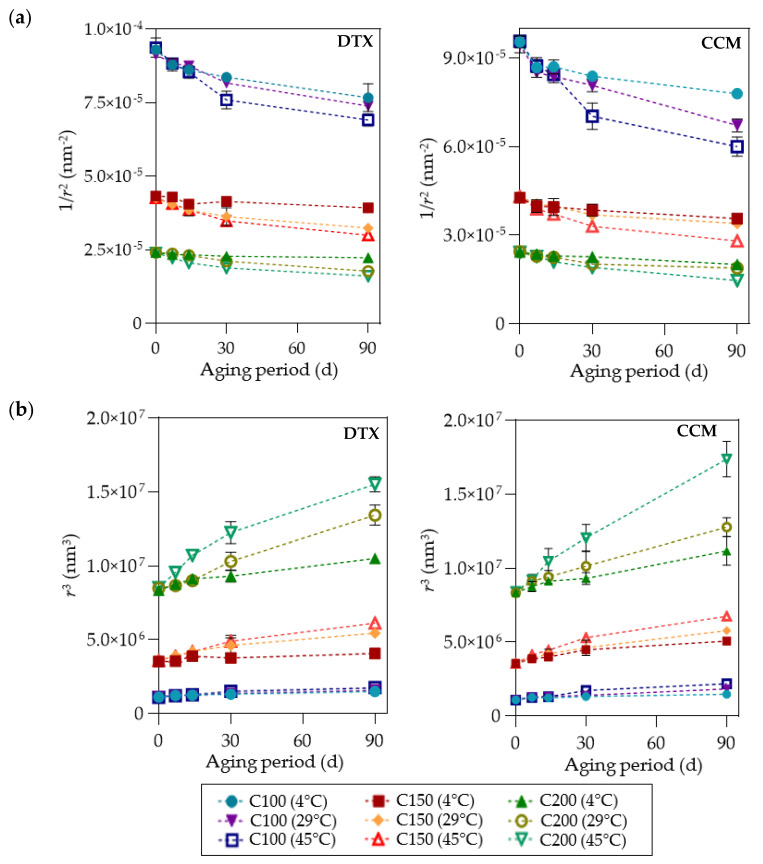
Graph of (**a**) coalescence (1/*r*^2^) and (**b**) Ostwald ripening (*r*^3^) of DTX- and CCM-loaded nanoemulsions against aging time (*t*) at different incubation temperatures. Error bars denote standard deviation (*n* = 3). DTX/D: Docetaxel; CCM/C: Curcumin; *r*: Mean particle size; d: Day.

The stability of the nanoemulsions produced in the current study at 4, 29, and 45 °C for 90 days of storage was determined. Only the nanoemulsion of 100 nm maintained a homogenous condition for up to 90 days at all temperatures assessed, while the D150 formulation remained stable for 90 days of storage at 4 °C. The D200 and C200 formulations only retained nanoemulsion homogeneity for up to five days. The findings were in close agreement with Stoke’s law, which stated that the rate of sedimentation or creaming decreases with increasing continuous phase viscosity [56]. Furthermore, the viscosity of the formulations in the present study improved as the particle size decreased, which therefore affected their stability. Several destabilizing mechanisms could determine the formulated nanoemulsion stability, including Ostwald ripening and coalescence. In this study, Ostwald ripening was the dominant destabilizing mechanism of the formulation with 200 nm particles at 4, 29, and 45 °C. The graph of *r*^3^ against (vs.) *t* in Figure 4 demonstrates a linear relationship with a higher Ostwald ripening rate due to the larger gradient compared to the 1/*r*^2^ vs. *t* (coalescence rate) plot. A similar trend was observed on the 150 nm formulation at 29 and 45 °C. Nonetheless, the Ostwald ripening rate was low at 4 °C for D150 compared to the C150 formulation. The results supported the physical stability evaluation findings, where the D150 sample remained unaffected after 90 days of storage at 4 °C, which was not observed in C150. According to Musa et al. and Usui et al., the Ostwald ripening rate of larger particle formulation could be enhanced in higher storage temperatures due to an increase in interfacial energy and solubility between smaller and larger droplets at high temperatures [57,58]. Accordingly, improved Ostwald ripening between nanoemulsion particles could be achieved by adjusting storage temperature.

### 3.5. Permeability Study

**Figure 5:**
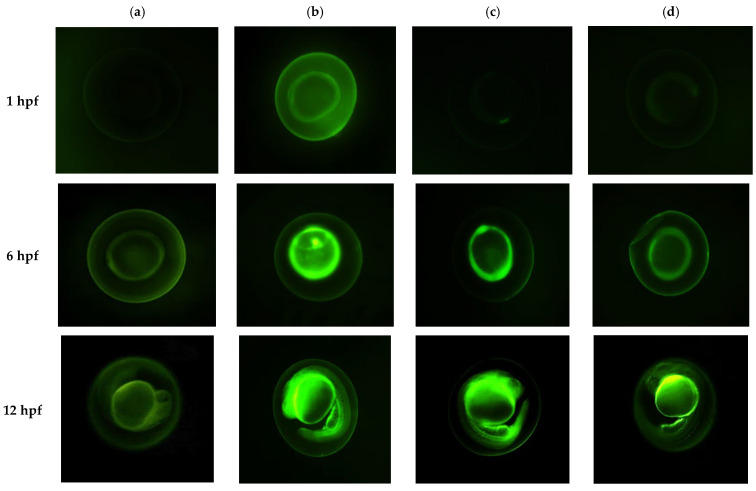
Fluorescence images of zebrafish (*Danio rerio*) embryos at the age of 1, 6, and 24 hpf (with chorion) after exposed to (**a**) 50 μg/mL fluorescein solution (control) and different sizes of nanoemulsions (**b**) NE 100, (**c**) NE 150, and (**d**) NE 200 containing 50 μg/mL fluorescein. The incubation was conducted at 28 ± 1 °C and the embryos were observed under an inverted fluorescence microscope and captured using CFI Plan Fluor 10× objective. Hpf: Hours post-fertilization, NE: Nanoemulsion.

Zebrafish embryos were selected to determine the permeability of the nanoemulsion formulations due to their suitability for microscopic imaging. The embryos also possess extremely low auto-fluorescence from fertilization to 24 h of post-fertilization (hpf) and provide clear fluorescent dye imaging passing through biological barriers (chorion), allowing permeability measurements [26]. The chorion possesses a multi-layered membrane containing glycoproteins as a major macromolecular component [59]. Based on Figure 5, the nanoemulsion with 100 nm particles permeated through the chorion as early as 1 hpf compared to the 150 and 200 nm systems and the control, which contained free fluorescein solution. The observations were consistent with Bali et al., where formulation with smaller particle size offered a large surface area and thus enhanced permeability [60]. Most of the fluorescein was detected localized to the inner spherical regions of the embryos at 6 hpf. The observation suggested that the formulation permeated into the yolk from the chorion layer and began partitioning into the cells during embryonic development. High fluorescence intensity was documented by the 100 nm formulation, followed by the 150 nm formulation. Conversely, the 200 nm and positive control (fluorescein solution) demonstrated the lowest fluorescence intensity. The findings in this study revealed that permeability significantly increased when nanoemulsion systems were employed instead of aqueous solutions, supporting the reports by Piazzini et al. and Shakeel and Ramadan [61,62].

**Figure 6:**
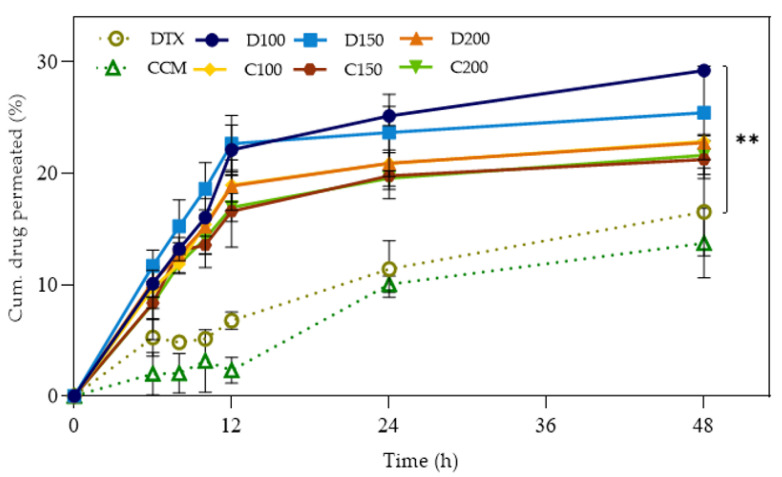
Drug release profiles of DTX- and CCM-loaded nanoemulsions using Franz-diffusion cell apparatus in simulated lung fluid (SLF) solution at pH 7.4. The analysis was carried out at 37 ± 1 °C for up to 48 h. Error bars denote standard deviation (*n* = 2) and asterisks denote statistically significant differences between groups (** *p* < 0.01). Cum: Cumulative; h: Hour; DTX/D: Docetaxel; CCM/C: Curcumin.

The Franz diffusion cells method was also used in assessing the drug release profile of the formulated nanoemulsion systems. A low-volume donor chamber with a semi-permeable membrane covered with 1% mucin solution was employed to imitate the limited lung lining volume and the mucous layer on the lung epithelium [63,64]. The DTX- and CCM-loaded nanoemulsions permeation profiles are illustrated in Figure 6. Similar permeation trends were recorded in the zebrafish and Franz-diffusion approaches, where formulations with smaller particles delivered the drug better than their larger counterparts. The phenomenon was due to the enhanced surface area-to-volume ratio from which the drugs were diffused from the oil core of the nanoemulsion system, hence, the faster drug delivery rate. The DTX-loaded formulation released the drug more rapidly than its CCM counterpart on account of differences in their ionization degree. The CCM ionization degree was slightly higher than the DTX at pH 7.4, leading to its inability to diffuse through the membrane effectively. Moreover, the discharge profile exhibited a biphasic pattern with a fast initial drug release within the first 12 h, followed by a sustained delivery up to 48 h. The observations might be due to the longer time required for the drug to completely diffuse through the oil droplet core and the surfactant interfacial layer before it is available in the dissolution medium, thus its release rate. Nevertheless, encapsulated drug deliveries were superior to free drugs solubilized in aqueous solutions. Consequently, the sustained release of encapsulated drug formulations could potentially extend exposure to the target site and improve efficacy in pulmonary applications.

### 3.6. Nanotoxicity Study

**Figure 7:**
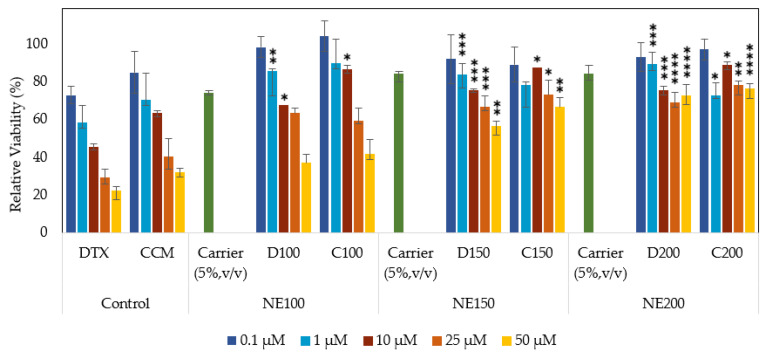
Cell viability of human lung fibroblast (MRC-5) cells analyzed by the MTT assay. Cells were incubated for 48 h at 37 ± 1 °C with different concentrations of controls and different size DTX- and CCM-loaded nanoemulsions (100, 150, and 200 nm). Error bars denote standard deviation (*n* = 3), and asterisks denote statistically significant differences between DTX- and CCM-loaded nanoemulsions with their respective control (**** *p* < 0.0001; *** *p* < 0.001; ** *p* < 0.01; * *p* < 0.05). DTX: Docetaxel; CCM: Curcumin; NE100: Nanoemulsion with a size of 100 nm; NE150: Nanoemulsion with a size of 150 nm; NE200: Nanoemulsion with a size of 200 nm.

The cell viability of human lung fibroblast (MRC-5) cells against DTX- and CCM-loaded nanoemulsions was determined using 3-[4,5-dimethylthiazol-2-yl]-2,5-diphenyl tetrazolium bromide (MTT) assay. This cell line was chosen as a model to assess the formulations’ cytotoxic potential on the most abundant normal cell type found in the lung interstitium [65]. Figure 7 illustrates the MRC-5 cell viability after 48 h post-exposure to the formulations and free form of unencapsulated drugs (control). In general, over 75% of the MRC-5 cell viability was recorded after exposure to 5% (*v*/*v*) blank nanoemulsions, indicating background toxicity absence in the carrier. A higher percentage of cell death was recorded by the free form of DTX (77%) and CCM (68%) in contrast to the DTX- and CCM-loaded nanoemulsions. The nanoemulsions exhibited dose- and size-dependent cytotoxicities. The cytotoxic effect was noticeable when the nanoemulsion with 100 nm particles was evaluated at a high drug concentration and showed no significant difference (*p* > 0.05) against the control. However, the C200, D200, C150, and D150 formulations significantly reduced the percentage of cell death when compared to their corresponding control. Consequently, encapsulated DTX and CCM in a nanoemulsion system could reduce toxicity on normal and healthy lung cells (MRC-5) compared to the free drugs.

## 4. Conclusions

Controlling the process parameters during the preparation is crucial to procure DTX- and CCM-loaded nanoemulsions with desired attributes for effective pulmonary delivery. The nanoemulsions were prepared with an overhead stirrer and high-shear homogenizer before being optimized via CCD as a multivariate modeling tool during process parameter studies. Both formulations exhibited significant condition interactions in determining the particle size and PDI (*p* < 0.0001) and a similar final reduced model to predict the response. The physicochemical characterization studies revealed that the mean particle size of the formulated nanoemulsions was almost identical to the targeted size (100, 150, and 200 nm) as predicted by the CCD models and exhibited preferable pH and viscosity characteristics for pulmonary administration. The nanoemulsion stability was reduced with increased particle size and storage temperature due to the Ostwald ripening process. The formulated nanoemulsions were also nebulized at a high drug amount and aerosol output (>98%). The aerosols’ aerodynamic diameters (VMD) also ranged from 4.6 to 5.5 μm, which is capable of deep lung deposition. Moreover, nanoemulsion formulations of 100 nm permeated faster than the larger particles (150 and 200 nm), leading to a high lung drug content, which is essential for lung-related disease local treatments with improved efficacy and reduced toxicity on normal cells.

## Figures and Tables

**Table 1:** The CCD coded independent variables used in RSM design.

Independent Variables	Coded Levels			
	−1	+1	−alpha	+alpha
Overhead stirrer rate (rpm), A	150	450	0	600
Overhead stirrer time (min), B	60	180	0	240
High shear rate (rpm), C	10,000	14,000	8000	16,000
High shear time (min), D	10	25	2.5	32.5

**Table 2:** Analysis of variance of the fitted quadratic and linear equations on particle size and PDI.

Source	DTX			CCM		
	Mean Square	*f*-Value	*p*-Value	Mean Square	*f*-Value	*p*-Value
Particle Size						
Model	2827.35	530.70	<0.0001	2045.99	226.76	<0.0001
A	1950.57	366.13	<0.0001	2116.13	234.54	<0.0001
B	924.68	173.57	<0.0001	1615.40	179.04	<0.0001
C	13,924.02	2613.59	<0.0001	12,415.13	1376.00	<0.0001
D	11,469.78	2152.92	<0.0001	7301.87	809.29	<0.0001
AB	128.53	24.12	0.0003	7.02	0.78	0.3916
AC	145.88	27.38	0.0002	141.37	15.67	0.0013
AD	41.04	7.70	0.0157	454.12	50.33	<0.0001
BC	294.27	55.24	<0.0001	16.20	1.80	0.2002
BD	855.32	160.55	<0.0001	77.88	8.63	0.0102
CD	8.99	1.69	0.2165	12.01	1.33	0.2667
A^2^	2270.97	426.27	<0.0001	480.00	53.20	<0.0001
B^2^	3025.98	567.99	<0.0001	451.68	50.06	<0.0001
C^2^	9.58	1.80	0.2028	2274.94	252.14	<0.0001
D^2^	1002.11	188.10	<0.0001	1072.56	118.87	<0.0001
Lack of fit	3.35	0.39	0.8839	10.83	2.00	0.2300
Pure error	8.50	-	-	5.41	-	-
PDI						
Model	0.014	78.25	<0.0001	0.029	29.81	<0.0001
A	<0.001	0.97	0.3339	<0.001	2.29	0.1437
B	<0.001	6.01	0.0219	0.001	10.81	0.0032
C	0.049	265.40	<0.0001	0.110	108.78	<0.0001
D	<0.001	33.49	<0.0001	<0.001	5.63	0.0263
Lack of fit	<0.001	2.96	0.1165	<0.001	1.03	0.5384
Pure error	<0.001	-	-	<0.001	-	-

DTX: Docetaxel; CCM: Curcumin; A: Overhead stirrer speed (rpm); B: Overhead stirrer duration (min); C: High shear speed (rpm); D: High shear duration (min).

**Table 3:** Response values for validation of DTX- and CCM-loaded nanoemulsion models.

Process Parameter				Particle Size (nm)			PDI		
A	B	C	D	Act.	Pre.	RSE (%)	Act.	Pre.	RSE (%)
DTX-loaded nanoemulsion									
300	55.5	14,000	26.0	103.5	100.1	3.40	0.256	0.248	3.23
200	81.0	13,000	20.0	151.9	150.0	1.27	0.271	0.283	4.24
200	21.5	11,000	10.0	203.6	200.1	1.75	0.345	0.359	3.90
CCM-loaded nanoemulsion									
300	56.0	14,000	19.7	99.3	100.0	0.70	0.185	0.194	4.64
300	62.0	12,000	10.0	148.6	150.1	1.00	0.273	0.282	3.19
300	14.0	10,000	10.0	197.9	200.0	1.05	0.358	0.367	2.45

A: Overhead stirrer speed (rpm); B: Overhead stirrer duration (min); C: High shear speed (rpm); D: High shear duration (min); Act.: Actual; Pre.: Predicted; RSE: Relative standard error; PDI: Polydispersity index.

**Table 4:** Physicochemical and aerodynamic characteristics of DTX- and CCM-loaded nanoemulsions.

Characteristics	D100	D150	D200	C100	C150	C200
Physicochemical characteristics						
Particle size (nm)	104.70 ± 0.44	152.40 ± 0.56	205.60 ± 2.45	101.23 ± 0.76	151.70 ± 1.74	202.33 ± 1.50
PDI	0.26 ± 0.02	0.26 ± 0.04	0.32 ± 0.03	0.18 ± 0.02	0.24 ± 0.03	0.34 ± 0.02
ζ-potential (mV)	−38.10 ± 1.41	−36.67 ± 0.45	−34.33 ± 1.20	−36.83 ± 1.25	−33.73 ± 0.51	−33.13 ± 0.77
pH	7.14 ± 0.01	7.14 ± 0.01	7.14 ± 0.01	7.13 ± 0.01	7.15 ± 0.01	7.14 ± 0.02
Viscosity (cP)	2.19 ± 0.13	2.06 ± 0.09	2.01 ± 0.07	2.01 ± 0.04	1.68 ± 0.04	1.59 ± 0.09
Aerodynamic characteristics						
Aerosol output (%)	98.32 ± 0.25	98.47 ± 0.11	98.53 ± 0.74	98.58 ± 0.22	98.84 ± 0.65	99.08 ± 0.51
Aerosol rate (g/min)	0.12 ± 0.01	0.15 ± 0.01	0.15 ± 0.01	0.16 ± 0.01	0.17 ± 0.01	0.19 ± 0.01
DV_10_ (μm)	2.43 ± 0.06	2.36 ± 0.06	2.07 ± 0.19	2.31 ± 0.05	2.11 ± 0.22	2.11 ± 0.17
DV_50_ (μm)	5.53 ± 0.26	5.11 ± 0.03	4.76 ± 0.37	5.03 ± 0.12	4.93 ± 0.04	4.63 ± 0.31
DV_90_ (μm)	11.73 ± 0.10	10.47 ± 0.18	10.50 ± 0.38	10.41 ± 0.57	10.74 ± 0.75	9.62 ± 0.92
D_[3,2]_ (μm)	4.52 ± 0.14	3.92 ± 0.56	3.91 ± 0.29	4.22 ± 0.07	3.99 ± 0.20	3.86 ± 0.29
D_[4,3]_ (μm)	6.42 ± 0.39	5.87 ± 0.02	8.50 ± 2.37	8.24 ± 4.41	5.80 ± 0.14	5.35 ± 0.38
GSD/Span	1.65 ± 0.11	1.59 ± 0.56	1.78 ± 0.12	1.61 ± 0.08	1.75 ± 0.21	1.61 ± 0.18

DV: distribution volume; GSD: geometric standard deviation; D: docetaxel; C: curcumin.

## Data Availability

All data relevant to the publication are included.
